# Modern Sociology

**Published:** 1903-05-23

**Authors:** 


### Modern Sociology.

### SHOP-GIRLS' HOURS.

It is much better to be a factory girl than a shop girl. So at least it would appear from the report given by Miss Irwin, Secretary to the Scottish Council for Women's Trades, before the Committee of the House of Lords on the Early Closing of Shops. It is true that the stately dames who condescend to serve us in large establishments are fairly well-off. At least they have regular meals, are provided with proper toilet-rooms, and are not ill-paid. But the girl who is sole assistant in a small shop has a bad time of it. The old-fashioned plan by which the owner of a shop lived behind it, and was assisted in its management by his wife and daughters, was much more wholesome than the modern system, which shuts the assistant into a draughty and ill-ventilated shop for a large number of hours each day, with perhaps no retreat for rest or comfort.

Miss Irwin has conducted an extensive investigation of shops in Glasgow, dealing especially with those which kept open after 10 P.M. These shops are opened in the morning between 8.30 and 9.30 as a rule, though a few are as late as 10 o'clock in taking down their shutters. The worst offenders in the matter of keeping late hours are dairies, fruiterers, newsagents, confectioners, tobacconists, restaurants, and ice-cream shops. In the poorer localities bakers and drapers were also found to keep open very late. The girls in these shops were often kept in them from 14 to 16 hours, and in some cases the hours worked at a stretch amounted to 17 hours. In the case of restaurants the hours might occasionally be even longer if one of the rooms happened to be let for any special entertainment. Moreover, the hours worked are longer than those in which the shop is open to the public, as it invariably takes some time after the place is closed to put away goods and make the place tidy. To add to the discomfort of these long hours of work, the temperature of the shop is often hot and vitiated. Miss Irwin found a temperature of from 75° to 80° Fahr. in some of the shops, when the outer air registered a temperature of only 40°. In many of these places there was no means of cooking food, no regular time for meals, and no sanitary conveniences. To aggravate the hardship of the situation, many of these girls live at a distance from the place where they work. If they are kept so late as to lose the last train or tram home, their tired limbs have often a long walk before them. There must be many whose condition at the end of their journey resembles that of the girl who told Miss Irwin, "When I get home, I just sit down and cry with fatigue."

The girls in tobacconists' shops are, perhaps, the most to be pitied of all, because they cannot be sure of having even a Sunday's rest. One girl said that the hours were from 8.30 A.M. to 11.15 P.M. for five days of the week, and to midnight on Saturdays, while the girls were expected to attend the shop on Sundays "when asked," which might happen every second or third Sunday. The girls in this shop were supposed to be free one evening in the week at seven or eight, but "could never count upon it." It was also understood that they were to have an hour and a half for meals each day, but they could not be sure of this respite either. Tobacconists who have shops in the neighbourhood of the theatres always keep open until these have closed, and though sometimes the proprietor himself relieves the shop-girl in the evening, or lets a male assistant do the work, this is not invariably the case.

Yet Miss Irwin does not seem to think that the proprietors should be blamed for this state of affairs, at least not the majority of them. Most of them assured her that they were willing to close early, "if only the other shops in the vicinity would do the same." This is an "if" in which there is not virtue, but much of the reverse. In the competition of trade the will of the minority gets more than its due meed of consideration. There seems to be no hope of simultaneous early closing by voluntary action, for shopkeepers who have tried it complain that there was always at least one defaulter, whose refusal to close at the same time as others kept all the rest from doing so. Sometimes shopkeepers who are under mutual agreement to close early have been known to watch their neighbours on the opposite side of the street to see who would put up the shutters first. No one was willing to risk being a minute before his neighbour. With such a spirit "early closing" is a farce. The shopkeepers seemed unanimous in desiring local compulsion to secure it, for voluntary union is apparently powerless.

It was suggested that in the poorer districts of any town, early closing might prove a hardship to buyers. The women of the working class go out to do their shopping late in the evening. On Saturday nights, when they do the bulk of their marketing for the week, they are to be seen about the streets, pricing and haggling, if not always buying, until close upon midnight. But Miss Irwin, who has a large acquaintance with the habits of this very class, declares that there is no need for special consideration for them. On the contrary, she affirms that it would often be a boon for them if the shops in their neighbourhood closed early on Saturdays. The men get their wages about one or two o'clock, and they would be more likely—at least so some of their wives affirm—to come straight home and give up the necessary money for the household, if there was a risk that if they were late, no food could be purchased for the morrow. As things are, they are too apt to spend the afternoon in a convenient public-house, and when at last they come home, the amount available for household necessities is far less than it otherwise might be. It is true that even women who receive their housekeeping money early do their shopping late, but that only means that they do not care to go out sooner than their neighbours. There is a social side to the marketing, and if convention fixes it at between ten and twelve on a Saturday night, you will find very few women go out earlier, although, if the shops were to close at eight, they would find it quite as easy to do their shopping before that time.

It was suggested that the shops might be allowed to keep open as long as the proprietors chose, but that the hours of the assistants should be fixed as they are in factories, and that the girls might work in relays. A maximum of 60 to 65 hours a week was suggested, instead of the present average of 90 to a 100, or even more. But as experience, especially with laundries, shows, it is very difficult to control places where shifts are worked. Unless an inspector could hang about the place in disguise all day, it would be practically impossible for him to see whether or not any assistant had worked beyond the legal limit. The absolute truth cannot be expected from a girl who knows that if she admits having worked longer than the law allows, she is likely to lose her situation. Working in shifts has always proved fruitful in evasion of the law, and as the shift system would probably permit of girls remaining in the shops they work in until very late at night, the plan might give rise to worse evils than those it stopped. Compulsory fixing of the hours of opening and shutting of shops seems to be the only practicable remedy.

